# Cryptococcal Meningoencephalitis 10 Years After Bone Marrow Transplant in a Patient With Multiple Myeloma

**DOI:** 10.7759/cureus.12094

**Published:** 2020-12-15

**Authors:** Gauthier Stepman, Jaya Sanapati, Kulveer Dabb, Debra Angelo, Johnathan Frunzi

**Affiliations:** 1 Internal Medicine, Medical Center of Trinity, Trinity, USA

**Keywords:** cryptococcus, cryptococcal meningoencephalitis, encephalitis, meningoencephalitis

## Abstract

Cryptococcosis is a fungal infection that mostly affects immunocompromised patients. Diagnosis is based on the detection of cryptococcal antigen in the cerebrospinal fluid (CSF) or serum. Antifungal resistance is emerging, making treatment difficult and long. We report a case of cryptococcosis in a patient with multiple myeloma, years after undergoing a bone marrow transplant. Symptoms were mild, and imaging studies were nonspecific. CSF analysis revealed positive cryptococcal antigen. The patient was started on the standard three-phase antifungal therapy and recovered.

## Introduction

Cryptococcosis is an invasive fungal infection caused by species within the genus *Cryptococcus *[1, 2]. Cryptococcosis was identified in 1891 but has only been recognized as a major health threat since the AIDS pandemic in the 1980s [1]. Cryptococcosis is predominantly seen in immunocompromised patients.

The most common cause of meningoencephalitis in immunocompromised patients is *Cryptococcus neoformans*. Some species of *Cryptococcus*, including *Cryptococcus gattii*, can cause life-threatening disease in immunocompetent individuals, however [3]. 

T-cells are often implicated in the defense against fungal species. Patients who have T-cell dysfunction, such as in HIV/AIDS, induction therapy for bone marrow transplant, proteasome inhibitor therapy, are at increased risk for cryptococcosis [4, 5].

Diagnosis of cryptococcosis is based on the clinical picture and detecting either the organism itself via India ink stain or the capsular antigen in serum or cerebrospinal fluid (CSF) [1, 2]. Treatment is difficult because of emerging resistance against antifungal drugs. Cryptococcosis often requires the use of multiple antifungal drugs, including fluconazole, liposomal amphotericin B (AmpB), and flucytosine [2].

## Case presentation

This case report describes a 70-year-old Hispanic male who presented to the hospital with a three-week history of headaches and subjective fevers. The patient’s daughter also described gait abnormalities and confusion. The patient described the headache as throbbing, retro-orbitally located. He denied any recent travel or close animal contacts. The patient provided a past medical history significant for multiple myeloma, diabetes mellitus type 2 with stage III diabetic nephropathy, and hypertension. He was diagnosed with multiple myeloma in 2007. He underwent a bone marrow transplant in 2010. The patient reported a three-year history of being treated with ixazomib, pomalidomide, and prednisone. On admission, the patient’s vital signs revealed a low-grade fever of 99.7 degrees Fahrenheit, heart rate of 78 beats per minute (BPM), blood pressure of 169/74 mmHg, and oxygen saturation of 95% on room air. His home medications were restarted. Throughout his hospital stay, the patient never developed a fever. Physical examination showed no meningeal signs, slight pallor of the conjunctivae, normal breath sounds without crackles, and a regular rate and rhythm. No gait abnormalities were noted in the hospital.

Laboratory results showed a normal leukocyte count, normal platelet count, and anemia with a hemoglobin concentration of 9.4 grams per deciliter (g/dL). His creatinine level on admission was 1.7 mg/dL. His CD4/CD8 ratio was found to be 0.59.

**Figure 1:**
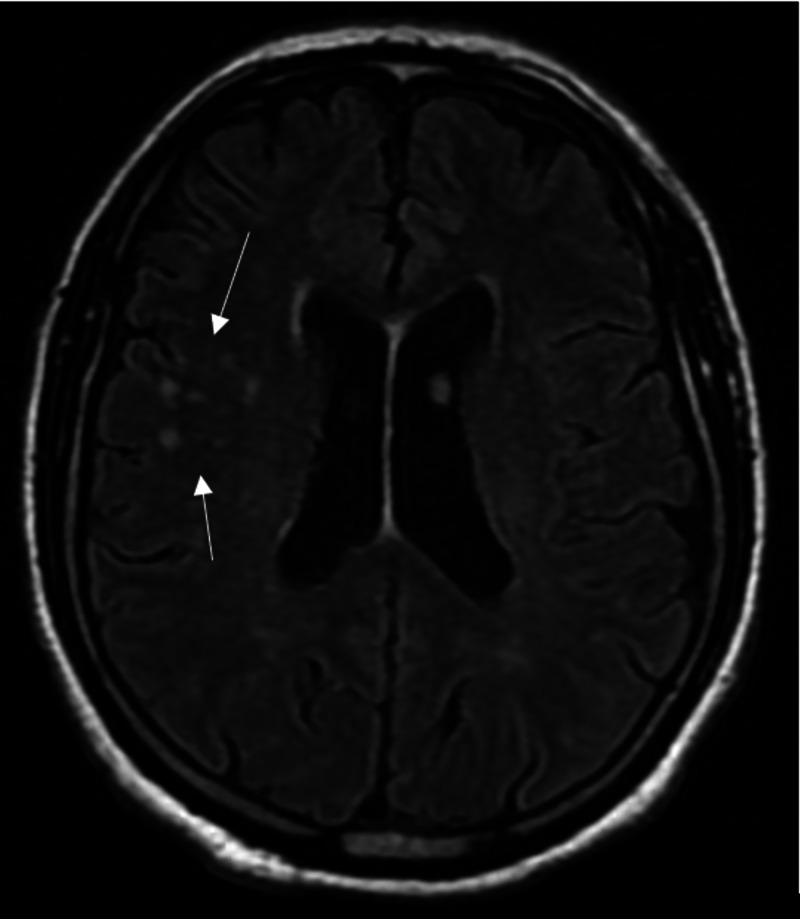
T2-FLAIR weighted magnetic resonance imaging (MRI) of the brain showing chronic cerebral demyelination (see arrows) FLAIR - fluid-attenuated inversion recovery

Upon admission, a computed tomography (CT) scan of the brain was obtained, which did not show any abnormalities. Subsequent magnetic resonance imaging (MRI) of the brain and spine was performed. The MRI revealed chronic cerebral demyelination and a normal spine (Figure 1).

The patient underwent a spinal tap with CSF analysis. The CSF was positive for *Cryptococcus *antigen with a 1:1280 titer. Other findings of the CSF analysis included a white blood cell (WBC) count of 27,000, with a lymphocytic predominance (65% lymphocytes), a total protein level of 73 mg/dL, and a glucose level of 60 mg/dL. Fungal culture identified *Cryptococcus neoformans* as a causative organism.

Diagnosis of cryptococcosis is based on the clinical picture and the detection of either the organism itself via India ink stain (Figure 1). The diagnosis of cryptococcal meningoencephalitis was made. The patient received a loading dose of 800 milligrams of fluconazole. We then started the patient on the standard treatment for cryptococcal meningoencephalitis, consisting of 5 milligrams per kilogram (mg/kg) of intravenous liposomal amphotericin B daily and 100 mg/kg of oral flucytosine. The patient was discharged after five days with a 10-week course of oral fluconazole at 400 mg/day and was instructed to continue fluconazole at 200 mg/day indefinitely after that. He reported significant improvement in his symptoms after starting treatment.

**Figure 2:**
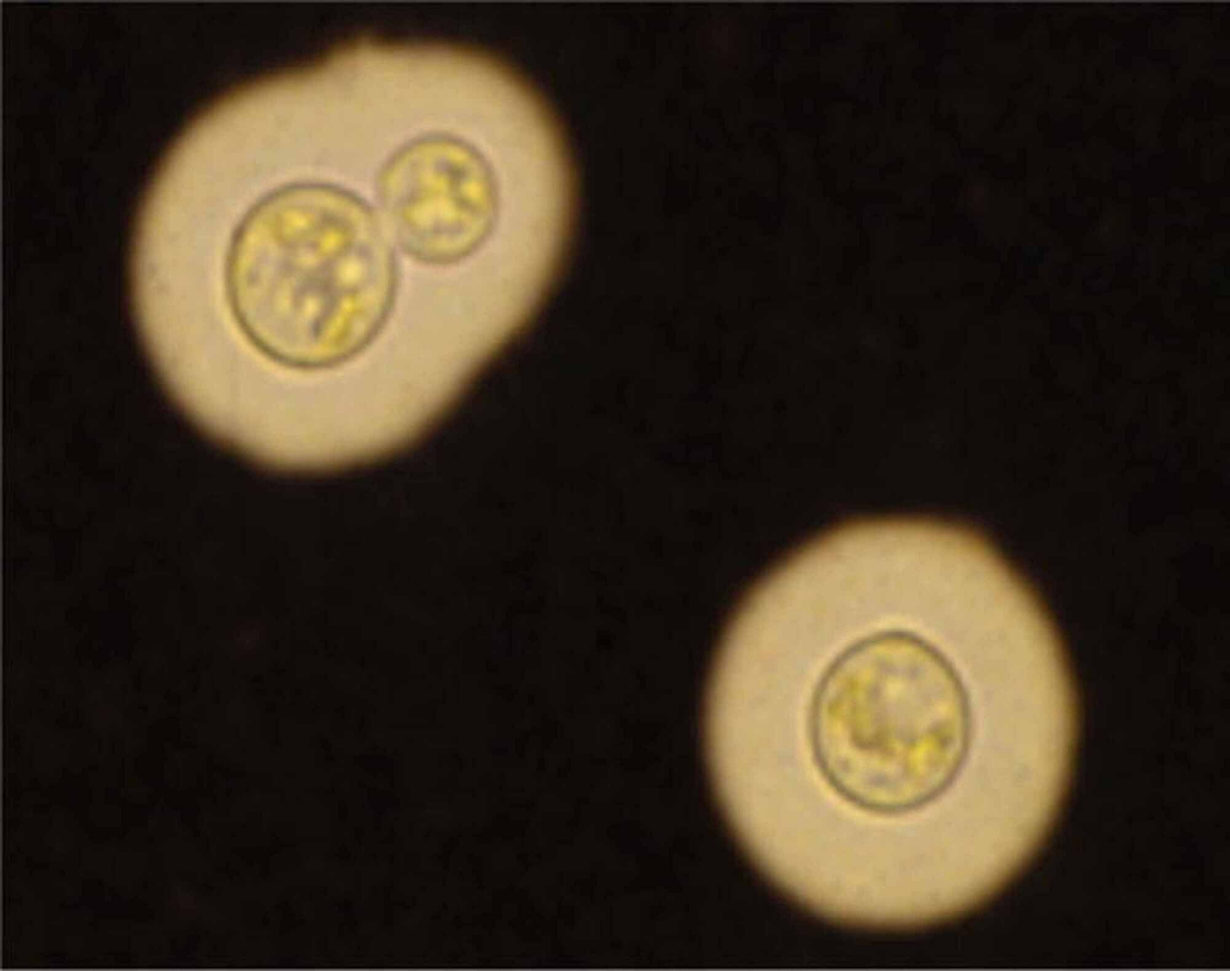
India ink staining showing encapsulated yeast Source: [2]

## Discussion

Immunocompromised patients are at increased risk for cryptococcosis. Meningoencephalitis is a life-threatening manifestation of disseminated cryptococcal infection. Case presentations of cryptococcal meningoencephalitis in patients with multiple myeloma are rare. We identified three cases of cryptococcal meningoencephalitis in patients with multiple myeloma [4-6]. Stem cell transplant was performed in two of the three cases. Time from transplant to presentation was four months in the case of Mendpara et al., and three weeks in the case of Fickweiler et al. [4, 5]. The case we present differs from the ones previously reported because our patient underwent a bone marrow transplant ten years before presentation. We were unable to identify any cases with a lag time longer than four months.

Our patient’s multiple myeloma is being treated with a proteasome inhibitor and an immunomodulator, which likely predisposed him for disseminated cryptococcosis. T-cell dysfunction is often present in cryptococcosis as it is the predominant component of host defense against the fungus [4]. Our patient’s absolute CD4 count was decreased, as was the CD4/CD8 ratio. This is similar to the case of Mendpara et al., where the CD4/CD8 ratio was altered due to high-dose cytotoxic antineoplastic therapy during autologous stem cell transplant [4]. In our case, the CD4/CD8 ratio was likely altered due to ixazomib treatment.

Presentations and imaging findings can be very nonspecific. Our patient had mild headaches, subjective fevers, and gait abnormalities. Fever and headache were also seen in the case of Fickweiler et al., while mainly neurologic symptoms were seen in the case of Shuku et al. [5, 6]. Our case differs from previously reported cases as our patient had no clear radiologic findings. Fickweiler et al. reported parenchymal and leptomeningeal involvement [5], while our patient only showed evidence of chronic demyelination (see Figure 2).

Current treatment guidelines for cryptococcosis consists of three phases: induction, consolidation, and maintenance. The induction phase is usually a combination of AmpB and flucytosine. Consolidation treatment is a prolonged course of high-dose fluconazole, typically 400 mg/day. The maintenance treatment is an indefinite time of fluconazole at 200 mg/day [2]. Mendpara et al. and Fickweiler et al. both followed this treatment regime [4, 5]. Their patients both improved clinically. The case of Shuku et al. was different in that the patient was critically ill and expired in the hospital despite AmpB and flucytosine treatment [6]. We opted to start with a loading dose of fluconazole at 800 mg/day as the patient already had underlying chronic kidney disease stage III. The patient and his daughter ultimately agreed to start the induction therapy, with good symptomatic improvement. The patient subsequently underwent consolidation therapy as an outpatient and is fairing well.

## Conclusions

Physicians need to be aware that cryptococcal meningoencephalitis is possible in patients diagnosed with multiple myeloma, even years after bone marrow transplant. It is essential to make a prompt diagnosis with detection of cryptococcal antigen in the CSF or visualization of the encapsulated yeast on India ink stain. Treatment with at least two antifungal agents should be initiated as soon as possible since cryptococcal meningoencephalitis can have devastating consequences.

## References

[1] May RC, Stone NRH, Wiesner DL, Bicanic T, Nielsen K (2016). Cryptococcus: from environmental saprophyte to global pathogen. Nat Rev Microbiol.

[2] Maziarz EK, Perfect JR (2016). Cryptococcosis. Infect Dis Clin North Am.

[3] Ma H, May RC (2009). Virulence in cryptococcus species (chapter 5). Adv Appl Microbiol.

[4] Mendpara SD, Ustun C, Kallab AM, Mazzella FM, Bilodeau PA, Jillella AP (2002). Cryptococcal meningitis following autologous stem cell transplantation in a patient with multiple myeloma. Bone Marrow Transplant.

[5] Fickweiler W, Aries MJH, Enting RH, Vellenga E, De Keyser J (2009). Cryptococcal cerebellitis after chemotherapy and autologous stem cell re-infusion in a patient with multiple myeloma. J Neurol.

[6] Sato S, Kambe E, Tamai Y (2019). Disseminated cryptococcosis in a patient with multiple myeloma treated with daratumumab, lenalidomide, and dexamethasone. Intern Med.

